# Genomic and Molecular Characterization of Miltefosine Resistance in *Leishmania infantum* Strains with Either Natural or Acquired Resistance through Experimental Selection of Intracellular Amastigotes

**DOI:** 10.1371/journal.pone.0154101

**Published:** 2016-04-28

**Authors:** Annelies Mondelaers, Maria P. Sanchez-Cañete, Sarah Hendrickx, Eline Eberhardt, Raquel Garcia-Hernandez, Laurence Lachaud, James Cotton, Mandy Sanders, Bart Cuypers, Hideo Imamura, Jean-Claude Dujardin, Peter Delputte, Paul Cos, Guy Caljon, Francisco Gamarro, Santiago Castanys, Louis Maes

**Affiliations:** 1 Laboratory for Microbiology, Parasitology and Hygiene (LMPH), University of Antwerp, Universiteitsplein 1, B-2610, Wilrijk, Belgium; 2 Instituto de Parasitologia y Biomedicina "Lopez-Neyra", Avda. Conocimiento S/N Parque Tecnológico Ciencias de la Salud, 18016, Granada, Spain; 3 Laboratoire de Parasitologie-Mycologie et Centre National de Référence des Leishmanioses, Centre Hospitalier Universitaire et Université de Montpellier 39, Avenue Charles Flahault, 34295, Montpellier, France; 4 Molecular Parasitology Unit (MPU), Institute of Tropical Medicine, Nationalestraat 155, B-2000, Antwerp, Belgium; 5 Advanced Database Research and Modeling (ADReM) research group, University of Antwerp, Middelheimlaan 1,2020, Antwerpen, Belgium; 6 Wellcome Trust Sanger Institute, Wellcome Trust Genome Campus, Hinxton CB10 1SA, Cambridge, United Kingdom; The Ohio State University, UNITED STATES

## Abstract

During the last decade miltefosine (MIL) has been used as first-line treatment for visceral leishmaniasis in endemic areas with antimonial resistance, but a decline in clinical effectiveness is now being reported. While only two MIL-resistant *Leishmania infantum* strains from HIV co-infected patients have been documented, phenotypic MIL-resistance for *L*. *donovani* has not yet been identified in the laboratory. Hence, a better understanding of the factors contributing to increased MIL-treatment failure is necessary. Given the paucity of defined MIL-resistant *L*. *donovani* clinical isolates, this study used an experimental amastigote-selected MIL-resistant *L*. *infantum* isolate (LEM3323). In-depth exploration of the MIL-resistant phenotype was performed by coupling genomic with phenotypic data to gain insight into gene function and the mutant phenotype. A naturally MIL-resistant *L*. *infantum* clinical isolate (LEM5159) was included to compare both datasets. Phenotypically, resistance was evaluated by determining intracellular amastigote susceptibility *in vitro* and actual MIL-uptake. Genomic analysis provided supportive evidence that the resistance selection model on intracellular amastigotes can be a good proxy for the *in vivo* field situation since both resistant strains showed mutations in the same inward transporter system responsible for the acquired MIL-resistant phenotype. In line with previous literature findings in promastigotes, our data confirm a defective import machinery through inactivation of the LiMT/LiRos3 protein complex as the main mechanism for MIL-resistance also in intracellular amastigotes. Whole genome sequencing analysis of LEM3323 revealed a 2 base pair deletion in the *LiMT* gene that led to the formation an early stop codon and a truncation of the LiMT protein. Interestingly, LEM5159 revealed mutations in both the *LiMT* and *LiRos3* genes, resulting in an aberrant expression of the LiMT protein. To verify that these mutations were indeed accountable for the acquired resistance, transfection experiments were performed to re-establish MIL-susceptibility. In LEM3323, susceptibility was restored upon expression of a *LiMT* wild-type gene, whereas the MIL-susceptibility of LEM5159 could be reversed after expression of the *LiRos3* wild-type gene. The aberrant expression profile of the LiMT protein could be restored upon rescue of the *LiRos3* gene both in the LEM5159 clinical isolate and a *ΔLiRos3* strain, showing that expression of LdMT is dependent on LdRos3 expression. The present findings clearly corroborate the pivotal role of the LiMT/LiRos3 complex in resistance towards MIL.

## Introduction

Visceral leishmaniasis (VL) is a tropical protozoan disease caused by *Leishmania donovani* and *L*. *infantum*. More than 500.000 new cases do occur annually [1] and control of this life-threatening condition has long been based on treatment with pentavalent antimonials (Sb^V^) [2]. To tackle the widespread Sb-resistance in the Indian subcontinent, miltefosine (MIL) was introduced in 2005 as first-line treatment for VL as part of the Kala-azar elimination program [3]. Although the phase-III trial that led to clinical approval of MIL in India demonstrated a 6-month cure rate of 94% [4], recent reports now indicate relapse rates of up to 20% [5,6]. At present, MIL is used in combination with paromomycin if a cold chain cannot be guaranteed. In Brazil, MIL-treatment of VL by *L*. *infantum* revealed a cure rate of only 43% [7]. To safeguard drug efficacy, the parasite-, host- and drug-related factors that contribute to MIL-treatment failure require further exploration. On the one hand, its pharmacokinetic properties [8] in addition to the long unsupervised treatment regimen [6,9] indeed put MIL at a considerable risk of selecting drug resistant parasites. While in the Indian subcontinent relapse after MIL-treatment could not yet be firmly linked to phenotypic resistance in *L*. *donovani* using the standard *in vitro* susceptibility assays [6,10], a potentially reduced MIL-susceptibility has been demonstrated in Brazilian *L*. *infantum* relapse isolates [7]. Rather surprisingly, only two *L*. *infantum* strains with definite natural MIL-resistance have been documented [11,12]. Given the overall paucity of MIL-resistant clinical field isolates, laboratory studies must generally rely on experimentally selected strains to explore MIL-resistance mechanisms and dynamics. It is noteworthy that most studies have used exposure of promastigotes to increasing MIL-concentrations, although selection of drug resistance on the more clinically relevant intracellular amastigote stage should be considered [13]. A common feature in MIL-resistant promastigotes is a decreased MIL-accumulation that is caused either by a defect in inward transport of MIL through inactivation of the *L*. *donovani* putative MIL-transporter (LdMT) [14] and/or its beta-subunit LdRos3 [15] or by an increased efflux mediated by the overexpression of ABC-transporter proteins [16].

In the present study, the experimentally selected MIL-resistant *L*. *infantum* strain LEM3323 [17] was subjected to an in-depth phenotypic and molecular characterization in direct comparison to its drug-susceptible wild-type parent counterpart. To further validate the *in vitro* intracellular amastigote resistance selection assay [13], the naturally MIL-resistant *L*. *infantum* clinical isolate LEM5159 was also investigated [12,18]. Characterisation of phenotypic resistance was based on *in vitro* amastigote and promastigote susceptibility and actual MIL-uptake, whereas next-generation sequencing explored the genomic basis of the resistant phenotypes in combination with functional validation of the detected mutations to confirm their contribution to the acquisition of resistance. Unravelling the genomic and molecular background of the laboratory experimental selected and clinical MIL-resistant *L*. *infantum* strains supports the relevance and validity of the *in vitro* amastigote model as a close proxy for the study of MIL-resistance in the field.

## Materials and Methods

### Chemical compounds

Miltefosine (hexadecylphosphocholine, Sigma-Aldrich, Diegem, Belgium) was dissolved in MilliQ water and stored at 4°C. The fluorescent analog of MIL (BODIPY-MIL) was kindly provided by L. Rivas (Madrid, Spain) [19]. [^14^C]MIL (1.33 MBq/mmol) was synthesized by Amersham Pharmacia Biotech (Buckinghamshire, United Kingdom). All other chemicals were of the highest quality and obtained from commercial vendors.

### *Leishmania infantum* strains

MHOM/FR/96/LEM3323 was obtained from a HIV-positive patient from the Languedoc area in Southern France and provided by CNRL, Montpellier, France. MHOM/FR/95/LEM3049 and MHOM/FR/2005/LEM5159 were isolated from the same patient, but with a ten-year time difference (provided by BRC-Leish, Montpellier, France). This patient had received several successive treatments with liposomal amphotericin B (AmB) [18] and MIL (personal communication Lachaud). Species identification was done by isoenzyme electrophoresis and pteridine-reductase 1 (PTR1) sequencing. Promastigote cultures were maintained at 25°C in haemoflagellate-modified minimal essential medium (HOMEM) (Gibco^®^, Life technologies, Ghent, Belgium) supplemented with 200 mM L-glutamine, 16.5 mM NaHCO_3_, 10% heat-inactivated fetal calf serum (iFCS), 40 mg/L adenine, 3 mg/L folic acid, 2 mg/L D-biotin and 2.5 mg/L hemin. Promastigotes of *L*. *infantum* ΔLiRos3 [20] and *L*. *donovani* ΔLdMT [15] null mutants were maintained at 28°C in RPMI-1640 medium (Gibco^®^, Life technologies, Ghent, Belgium) supplemented with 10% iFCS. The number of passages was kept as low as possible to maintain parasite virulence.

### Laboratory animals

Laboratory animals were used to provide primary peritoneal macrophages for the *in vitro* work. Female Swiss mice were obtained from Janvier (Le Genest Saint Isle, France) and kept on a regular rodent diet and given drinking water *ad libitum*. Twenty-four hours after intraperitoneal stimulation with a 0.2% potato starch solution, animals were euthanized with a CO_2_ overdose. Primary peritoneal macrophages (PPM) were collected as previously described [21].

### Drug susceptibility assays

The *in vitro* MIL-susceptibility was determined at both promastigote and intracellular amastigote level as previously described [22]. In brief, MIL IC_50_-values of log-phase promastigotes were assessed by exposing the parasites to serial two-fold MIL-dilutions. After 72h incubation, viability testing was performed by adding resazurin and measuring the fluorescence by spectrophotometry (Tecan^®^, GENios). Evaluation of the susceptibility of intracellular amastigotes was done after five days of MIL-treatment and microscopic determination of the reduction in amastigote burdens per cell upon Giemsa-staining.

### Experimental selection of MIL-resistance

The parent clone of *L*. *infantum* (MHOM/FR/96/LEM3323 Cl-4) was subjected to resistance selection on intracellular amastigotes, as previously described [13,17]. The resistance selection cycles were repeated until the arbitrarily set cut-off value of 15 μM for MIL-resistance on amastigote level was achieved [23]. The selected resistant population was cloned again using the micro-drop method and one clone was randomly selected to perform all experiments [13]. A stable MIL-resistant phenotype (LEM3323-MIL) had already been experimentally selected on intracellular amastigotes of the parent LEM3323 strain [17].

### Whole-genome sequencing

Next generation sequencing was performed in collaboration with the Center of Medical genetics (CMG, University of Antwerp, Belgium) and the Welcome Trust Sanger Institute (WTSI, Hinxton, United Kingdom). DNA was isolated from a pellet of stationary-phase promastigotes of *L*. *infantum* strains LEM3049, LEM5159, LEM3323 and LEM3323-MIL using QIAamp DNA Mini kit (Qiagen, Netherlands). DNA concentration was measured by Qubit^®^ fluorimeter using the Qubit^®^ dsDNA BR Assay Kit (Thermo Scientific, Belgium). Libraries of LEM3323 and LEM3323-MIL were prepared with the Nextera XT sample prep kit (Illumina) and sequenced using the Illumina Miseq at CMG. LEM3049 and LEM5159 were sequenced according to Shaw et al. (*in press*) [24] at WTSI and deposited in the European Nucleotide Archive with the accession numbers ERS340107 and ERS340108 respectively. Reads (with an average 32X coverage) were aligned to the *L*. *infantum* JPCM5 reference genome (TriTrypDB version 8.0) with Bowtie2 [25–27] and variants were called with Samtools Mpileup [28]. Using a Python script, variants were selected with a read coverage of at least 5 for each strain, a variant quality of at least 50 and mapping quality of minimum 30. Alleles that differed between the MIL-susceptible LEM3323 and MIL-resistant strain LEM3323-MIL were retained and manually verified in IGV [29]. Chromosome copy number was determined by measuring the median read depth of each chromosome *d*_*i*_, and obtaining the median depth of the 36 chromosomes *d*_*m*_. The somy status of each chromosome was defined by *d*_*i*_*/d*_*m*_ and the biological ploidy value was defined as 2*d_*i*_*/d*_*m*_ for a strain whose major ploidy status was diploid.

### DNA constructions and generation of transfected parasites

Generation of the *Leishmania* expression vectors containing *LiMT* and *LiRos3* and the *LiMT/GFP* fusion protein were previously described [14,20]. The *LiMT*^*E926Q*^*GFP* mutant construct with GFP at the C-terminus was developed using the primers P1 (5' GAAGATGCCCTGCTGCAGCGGCCGAAGCTGTAC 3') and P2 (5' GTACAGCTTCGGCCGCTGCAGCAGGGCATCTTC 3') and the QuikChange site-directed mutagenesis kit (Agilent Technologies, Diegem, Belgium) using *LiMT-GFP* as template. Promastigotes (3 x 10^7^) were transfected by electroporation (450 V, 500 μF) and the *LiMT* and *LiRos3* transfects were selected with 200 μg/ml hygromycin [14]. The transfected ΔLdMT null mutants were selected with 200 μg/ml geneticin. The expression level of GFP-fused proteins in *LiMT-GFP* and *LiMT*^*E926Q*^*GFP* transfected parasites was determined by flow cytometry in a FACScan flow cytometer (Becton-Dickinson).

### Determination of intracellular MIL-accumulation

Preliminary work on differences in MIL-uptake between resistant and susceptible parasites was done using BODIPY-MIL, a highly fluorescent and photostable MIL-analogue that allows visualization of MIL-uptake in both extracellular promastigotes and intracellular amastigotes [19]. The intracellular accumulation of MIL in transfected *Leishmania* promastigotes was quantitatively evaluated by measuring intracellular [^14^C]MIL-accumulation [30]. Briefly, 2 x 10^7^ promastigotes were incubated with 0.09 μCi/ml [^14^C]MIL (2.5 μM) for 60 min at 28°C in culture medium. The parasites were then washed with ice-cold 1% BSA-PBS for removal of the drug fraction bound to the outer plasma membrane, followed by a second wash. Both protein concentration and counts per minute were determined.

### Expression level analysis

Promastigotes (3 x 10^7^ cells/ml) were harvested by centrifugation and washed three times in cold PBS. Parasites were suspended in PBS supplemented with protease inhibitor cocktail (Sigma-Aldrich, Diegem, Belgium) and solubilised with lysis buffer containing 50 mM Tris, 150 mM NaCl and 2% dodecyl maltoside (DDM). Protein samples were fractionated by SDS-polyacrylamide gel electrophoresis using standard conditions and electro-transferred onto Immobilon-P membranes (Merck Millipore, Belgium). Immunodetection was performed with 1:300 dilution of rabbit anti-LdMT antibody and 1:1000 dilution of rabbit anti-LdRos3 antibody [31] in PBS containing 0.1% Tween 20 and 0.1% BSA; α-tubulin was detected using a 1:12500 dilution of a mouse monoclonal anti-α-tubulin antibody (Sigma-Aldrich, Diegem, Belgium); the GFP-fused proteins were detected using a 1:5000 dilution of a rabbit anti-GFP antibody (Life technologies, Ghent, Belgium). After washing, membranes were incubated with 1:5000 dilution of horseradish peroxidase-conjugated secondary goat anti-rabbit or anti-mouse immunoglobulin G (Dako, Agilent Technologies, Belgium). Signals were detected by the ECL chemiluminescent substrate (Thermo Scientific^TM^ Pierce^TM^ Protein Biology, Life Technologies, Belgium).

### Ethics statement

This study using laboratory rodents was carried out in strict accordance to all mandatory guidelines (EU directives, including the Revised Directive 2010/63/EU on the protection of Animals used for Scientific Purposes that came into force on 01/01/2013, and the declaration of Helsinki in its latest version) and was approved by the ethical committee of the University of Antwerp, Belgium (UA-ECD 2010–17).

### Statistical analysis

Statistical comparisons between groups were performed using Student’s *t-*test. Differences were considered significant at a level of *p* < 0.05.

## Results

### *In vitro* MIL-susceptibility

Promastigote and amastigote susceptibilities of the parent and the derived resistant lines are summarized in Table 1. Noting that an infection ratio of only 2 parasites per macrophage was used, both strains showed very high infectivity with an average infection index of 13.4 ± 1.7 intracellular amastigotes/macrophage at 24 hours post-infection for the WT and of 7.3 ± 1.9 for the MIL-R strain. Both promastigotes and amastigotes of LEM5159, isolated from an HIV-infected patient after ten years of therapeutic intervention [18], confirmed a stable MIL-unresponsiveness for at least twenty successive passages without drug pressure [12]. LEM3049 that was collected from the same patient before receiving several MIL-treatment rounds showed full MIL-susceptibility (Table 1).

**Table 1:** Susceptibility to miltefosine (MIL) of the different *L*. *infantum* strains.

Strain	Intracellular amastigotes IC_50_ (mean ± SEM)	Promastigotes IC_50_ (mean ± SEM)
LEM3323	2.3 ± 0.5	5.3 ± 0.3
LEM3323-MIL	>20.0	>40.0
LEM3323-MIL *+ LiMT*	3.0 ± 1.0	2.4 ± 1.2
LEM3049	0.8 ± 0.4	5.6 ± 1.1
LEM5159	> 20.0	> 40.0
LEM5159 + *LiRos*3	0.5 ± 0.3	3.8 ± 1.3
LEM5159 + *LiMT*	> 20.0	> 40.0
Δ*LdMT*	> 20.0	> 40.0
Δ*LdMT* + *LiMTGFP*	3.5 ± 2.3	12.8 ± 0.8
Δ*LdMT* + *LiMT*^E926Q^GFP	4.3 ± 1.6	16.1 ± 0.8

Li: L. infantum; Ld: L. donovani

The average IC_50_-values (μM) ± the standard error of the mean (SEM) of intracellular amastigotes and promastigotes are shown. LEM3323 was subjected to *in vitro* MIL-resistance selection, whereas LEM5159 had a natural MIL-resistant phenotype. The data shown are the result of three independent tests run in duplicate.

### Whole-genome sequencing

To unravel the underlying mechanisms responsible for the resistant phenotype, whole-genome sequencing was used to compare LEM5159 and LEM3323-MIL with the pre-treatment isolate LEM3049 and the wild-type (WT) LEM3323 (Table 2).

**Table 2:** Coding sequence mutations within the LiMT and LiRos3 MIL-transporting complex, identified in the *L*. *infantum* MIL-resistant strains.

Strain	*LiMT* gene (LinJ.13.1590)	*LiRos3* gene (LinJ.32.0540)
LEM3323		
LEM3323-MIL	INDEL CCA**CA** to CCA (619572)	
LEM3049		
LEM5159	codon change GAG to CAG (617837)	INDEL TTTTT**T**A to TTTTTA (189831)

LEM3323-MIL: from the genome sequence point of view, LEM3323-MIL was rather similar to its WT parent: in total, between the two strains, we found only 40 single nucleotide polymorphisms (SNPs) that passed the quality filters: 7 variants were present within a coding region and passed manual verification in IGV. Only one gene was changed from a homozygous reference sequence to a homozygous variant (homozygous, non-synonymous single nucleotide polymorphism SNP) (indel LinJ.13.1590 or *LiMT*). The other 6 changes were still heterozygous for the reference allele (and hence should still produce approximately 50% of the functional protein) or comprised synonymous mutations (see S1 Table). The homozygous two base pair (CA) deletion in the *LiMT* transporter gene on position 1037–1038 of the gene (equivalent to position 619572–619573 within chromosome 13) caused a shift of the reading frame generating a stop codon after 369 of the 1097 amino acids. No variant was found in the *LiRos3* gene (LinJ.32.0540) coding sequence or in 5’ or 3’UTRs. From the karyotype point of view, both LEM3323-MIL and the WT strain were aneuploid with a decrease in copy number of four chromosomes (1, 2, 9, 12) in LEM3323-MIL, the maximal being observed for chr1 (-1 copy, S1 Fig, panel A).

LEM5159: The genome sequence of LEM5159 was drastically different from that of the putative pre-treatment isolate LEM3049, with 11,570 SNPs between both lines: possible traces of LEM5159-specific reads were searched for in the sequencing reads of LEM3049, but were not encountered. Considering the average 32x coverage achieved in our sequencing, this indicates that the patient was re-infected with LEM5159 during his long clinical history or that LEM5159 was present at the onset at a proportion lower than about 1/32 (vs LEM3049). Particular attention was given to the two genes incriminated in the experimental MIL-resistance: (i) a single SNP was detected in the aminophospholipid translocase *LiMT* gene resulting in an E to Q substitution at codon 926 (GAG → CAG) and (ii) a frameshift mutation (deletion of T base at base 103 of the gene) in the *LiRos3* gene causing an early stop codon at amino acid 49. Both isolates were aneuploid with more changes than in the experimental pair described above (S1 Fig, panel B): eight chromosomes (2, 8, 10, 20, 22, 23, 29, 35) showed a higher ploidy in the MIL-resistant isolate LEM5159 (vs LEM3049), the largest being for chromosome 23 (+2 copies, S1 Fig, panel B); two chromosomes (12, 31) showed a lower ploidy in LEM5159, the largest (-2 copies, S1 Fig, panel B) in chromosome 12.

### Cell transfections and reconstitution of the MIL-susceptible phenotype

To study the role of the mutations found in the *LiMT/LiRos3* genes in the loss of translocase activity and the acquisition of the resistant phenotypes, transfections with expression vectors containing the gene of interest were performed, followed by drug susceptibility testing on promastigotes and intracellular amastigotes (Table 1).

LEM3323-MIL: Transfection with a *LiMT* gene obtained from a wild-type *L*. *infantum* strain resulted in rescue of the resistant phenotype in both parasite stages, confirming that the single indel found in the *LiMT* gene fully accounts for the intrinsic resistance phenotype of the LEM3323-MIL strain.

LEM5159: To verify if the truncation in the *LiRos3* gene is accountable for the acquired resistance, transfection with *LiRos3* gene obtained from a wild-type *L*. *infantum* strain re-established MIL-susceptibility in the transfected strain (LEM5159 + *LiRos3*) in both parasite stages (Table 1). However, transfection of LEM5159 with a *LiMT* wild-type gene obtained by PCR from a wild-type *L*. *infantum* strain was not able to rescue susceptibility since both proteins are necessary in the MIL- transporter complex [15]. The specific contribution of the *LiMT* mutation (E926Q) in the acquisition of MIL-resistance was further analysed. A plasmid containing the mutation present in the *LiMT* gene *(LiMT*^*E926Q*^*GFP*) was introduced in a ΔLdMT strain using the ΔLdMT + *LiMT* as a control, after determining that the level of wild type LiMTGFP and LiMT^E926Q^GFP were similar across the two transfected strains. MIL-susceptibility was checked *in vitro* comparing both transfected lines and both parasite stages and no differences were found. Altogether, these results suggest that the point mutation E926Q does not affect the function of LiMT and that the SNP found in the *LiMT* gene is not involved in the reduction of MIL-susceptibility of LEM5159.

### Determination of intracellular MIL-accumulation

**Fig 1:**
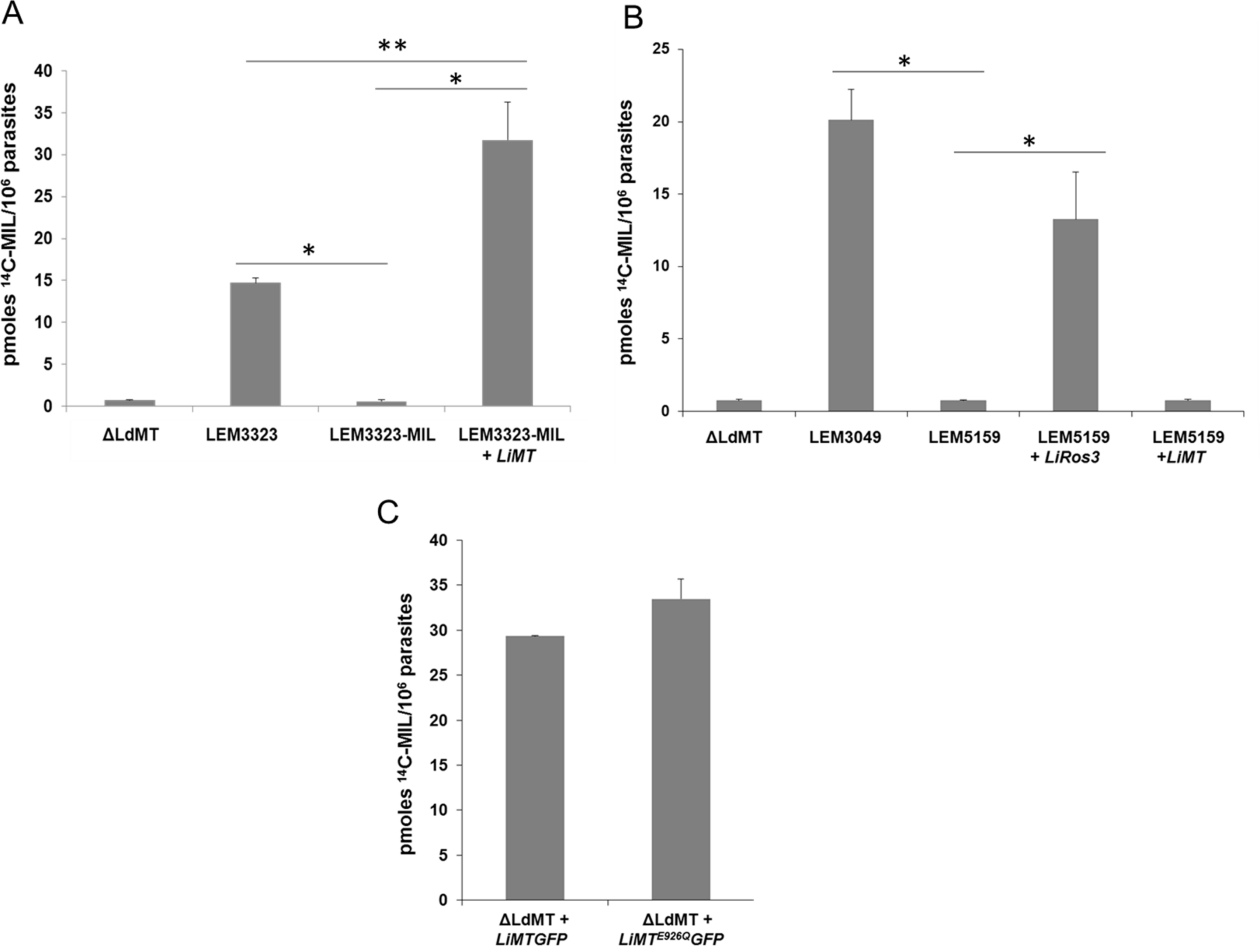
Determination of intracellular MIL-accumulation. Uptake of [^14^C]MIL by *L*. *infantum* and *L*. *donovani* promastigotes was measured after incubation for 60 min at 28^◦^C (A-C). Results are expressed as the mean ± the standard deviation of three independent experiments in duplicate. **(A)** *L*. *donovani* Δ*LdMT* line *L*. *infantum* LEM3323, LEM3323-MIL (LEM3323MIL), *LiMT*-transfected LEM3323-MIL (LEM3323-MIL + *LiMT*), **(B)** LEM3049, LEM5159, *LiRos3*- and *LiMT*-transfected LEM5159 (LEM5159 + *LiRos3*; LEM5159 + *LiMT*) and **(C)** *L*. *donovani* Δ*LdMT* promastigotes transfected with *LiMT* GFP and with *LiMT*^E926Q^GFP. Significant differences were determined using the Student's *t* test (*, *p* < 0.001, ** *p* < 0.005).

Preliminary experiments with BODIPY-MIL in promastigotes (S2 Fig) and intracellular amastigotes (S3 Fig) already indicated marked differences between MIL-sensitive and MIL-resistant parasites in terms of uptake. To confirm these preliminary data, uptake of [^14^C]MIL was evaluated in the WT parent and MIL-resistant strains and in the transfected strains (Fig 1).

LEM3323-MIL: Given the presence of the indel in the *LiMT* gene, internalization of [^14^C]MIL was evaluated in the transfected strain LEM3323-MIL + *LiMT* to study whether the MIL-internalization was restored within 60 min incubation. While LEM3323-MIL showed a strong reduction in [^14^C]MIL accumulation compared to the MIL-susceptible parent strain, recovery of MIL-uptake in the transfected strain was complete (Fig 1A) and identifies the involvement of the major truncation in *LiMT* gene in the acquisition of resistance in LEM3323-MIL.

LEM5159: The same experiment was performed on LEM3049, LEM5159 and the transfected strains LEM5159 + *LiRos3* and LEM5159 + *LiMT* (Fig 1B). Since LEM3049 was isolated from the patient before the start of MIL-treatment, its extensive MIL-accumulation confirms the presence of an intact *LiMT/LiRos3* transporter complex. In contrast, LEM5159 showed almost no MIL-uptake while transfection with *LiRos3* was able to rescue the defect in MIL-internalization (Fig 1B) while *LiMT*-transfected LEM5159 parasites were still defective in MIL-uptake (Fig 1B). To examine the contribution of the mutation *LiMT*^E926Q^*GFP* in the decreased MIL-susceptibility profile of LEM5159, MIL-internalization was also measured in the *LiMT*^E926Q^*GFP* transfected Δ*LdMT* line (Fig 1C). The Δ*LdMT* + *LiMT*^E926Q^*GFP* line showed a high ability to take up MIL, with no significant difference to the control line Δ*LdMT* + *LiMTGFP* (Fig 1C), endorsing that the E926Q mutation in the *LiMT* gene is not responsible for the resistant phenotype of LEM5159.

### Expression level analysis

**Fig 2:**
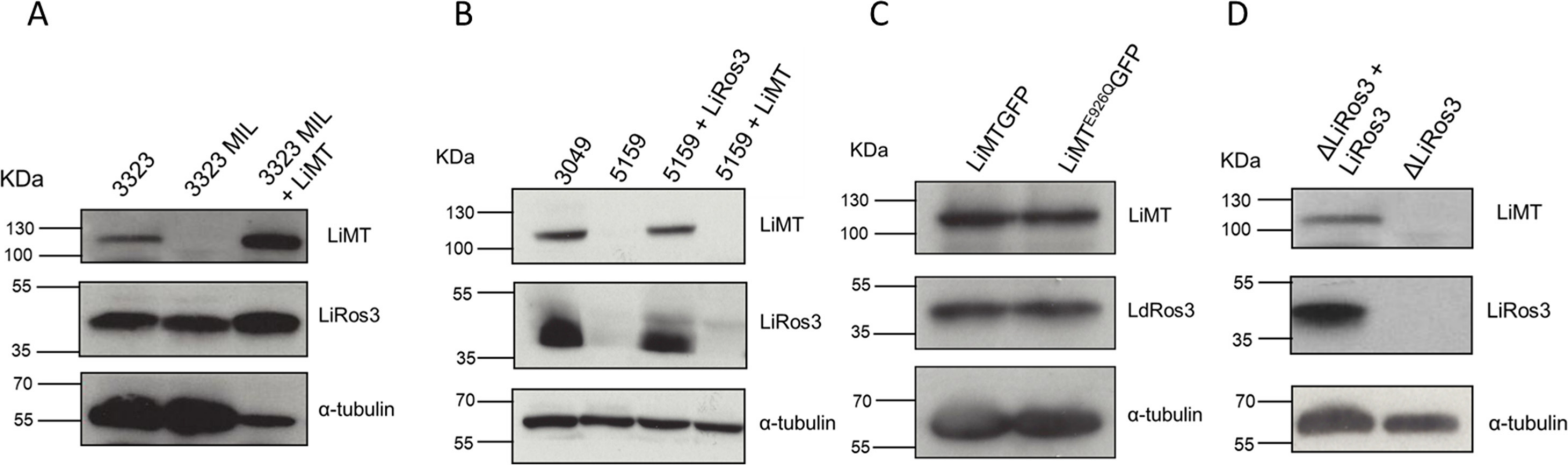
Analysis of the expression levels of the MIL-translocation machinery in different *Leishmania* strains. Extracts from (A) *L*. *infantum* LEM3323 (3323), LEM3323-MIL (3323MIL), *LiMT*-transfected LEM3323-MIL (LEM3323-MIL + *LiMT*), (B) LEM3049, LEM5159, *LiRos3*- and *LiMT*-transfected LEM5159 (LEM5159 + *LiRos3*; 5159 + *LiMT*), (C) *L*. *donovani* Δ*LdMT* promastigotes transfected with LiMT GFP and with LiMT^E926Q^GFP and (D) Δ*LiRos3* and Δ*LiRos3* + *LiRos3* lines were subjected to SDS/PAGE and immunoblotted with the rabbit polyclonal anti-*LdM*T and anti-*LdRos3* antibodies. Anti-α-tubulin monoclonal antibody was used as a probe for a protein loading control.

LEM3323-MIL: To validate the impact of the truncation present in the *LiMT* gene on *LiMT* and *LiRos3* protein expression levels, western blot experiments were performed using anti-*LdMT* and anti-*LdRos3* antibodies (Fig 2). Expression levels of α-tubulin was used as a probe for protein loading control. LEM3323-MIL showed similar expression levels of *LiRos3* as LEM3323 but no expression of the *LiMT* protein, clearly demonstrating that the absence of the LiMT protein renders the wild-type LEM3323 refractory to MIL (Fig 2A).

LEM5159: The expression levels of LiMT and LiRos3 protein of LEM3049, LEM5159 and the *LiMT*- and *LiRos3*-transfected lines were analysed by western blotting (Fig 2B). The MIL-susceptible LEM3049 displayed a clear band for both LiMT and LiRos3 proteins, indicating the presence of a fully functional inward transporter complex. LEM5159 was defective in the expression of both LiMT and LiRos3 protein. Surprisingly, protein expression of *LiMT* and *LiRos3* was restored after transfection with *LiRos3* gene implying that the activity and location of both proteins depend upon one another (Fig 2B). Since *LiRos3* protein was seriously affected by the frameshift mutation, expression of *LiMT* did not occur, as illustrated in the *LiMT*-transfected LEM5159 line (Fig 2B). Hence, *LiRos3* protein is necessary to maintain correct levels of LiMT protein, as is demonstrated in the Δ*LiRos3* strain which revealed no expression of both proteins (Fig 2D). Demonstrating that Δ*LdMT* + *LiMT*^E926Q^*GFP* and Δ*LdMT* + *LiMTGFP* lines had similar levels of both proteins (Fig 2C), MIL-susceptibility and internalization assays were performed to fully corroborate that E926Q substitution in *LiMT* does not affect the MIL uptake (Fig 2C).

## Discussion

The current increasing trend for MIL-treatment relapses of *L*. *donovani* in endemic areas in the Indian subcontinent [5,6] in combination with the fact that intrinsic phenotypic resistance in isolates from relapse patients has not unequivocally been demonstrated using the standard *in vitro* susceptibility laboratory assays [6,10,32] triggers the need for in-depth exploration of the phenotypic and genotypic characteristics of MIL-resistant *Leishmania* species/strains. Facing the fact that MIL-resistant *L*. *donovani* patient isolates are not yet available while two MIL-resistant *L*. *infantum* strains have already been documented in HIV co-infected patients [11,12] directed our focus towards *L*. *infantum*. While resistance has mostly been studied in laboratory-selected promastigotes, it should be recognized that focusing such research on the intracellular amastigote stage is definitely more relevant. After having developed such an alternative resistance selection assay on intracellular amastigotes [13], our research succeeded in experimentally selecting full MIL-resistance in an *L*. *infantum* field isolate [17,33], adding the particular advantage that the parent drug-susceptible LEM3323 and the derived MIL-resistant mutant LEM3323-MIL could be directly compared for phenotypic and genotypic analysis. Although the LEM3323-MIL strain achieved resistance within five successive cycles of drug pressure, *in vitro* generation of additional MIL-resistant strains of *L*. *donovani* and *L*. *infantum* at amastigote level has proven to be quite challenging [12,17] and suggests that MIL-resistance may not that easily be selected in the field as originally anticipated based on pharmacokinetic [8] and treatment compliance [6,9] considerations. The MIL-resistant clinical field isolate LEM5159 was included as a crosscheck for the validity of the *in vitro* amastigote resistance selection model. LEM5159 was isolated from a HIV-positive patient who relapsed 8 times and received successive cycles of MIL during a five-year period [34]. It is well known that immunocompromised patients develop a chronic infection with poor responsiveness to repetitive MIL-treatments [35,36], also due to the absence of an adequate host response [37]. It is still an open question whether such conditions may also lead to full MIL-resistance in *L*. *donovani* or *L*. *infantum* in an immune-competent patient.

Unresponsiveness of LEM3323-MIL and LEM5159 was established by *in vitro* susceptibility assays on promastigotes and intracellular amastigotes (Table 1). Research on MIL-resistant promastigotes already suggested that decreased MIL-accumulation is a plausible resistance mechanism that is achieved by a defect in MIL uptake through inactivation of the *LdMT* transporter [30] and/or its beta-subunit *LdRos3* [15]) or by an increased efflux mediated by the overexpression of ABC-transporter proteins [16]. To check if the observed decreased MIL-accumulation acquired at intracellular amastigote level had indeed the same mechanistic basis as described for promastigotes, whole genome sequencing was conducted to identify mutations involved in MIL-resistance.

Previously, genetic analysis of a natural MIL-resistant *L*. *infantum* strain identified a SNP in the *LiMT* gene, pointing to a correlation between the mutation and the reduced MIL-susceptibility [11]. Unfortunately, no functional experiments on this strain were performed to clarify the exact mechanism of resistance, highlighting the importance of the present study that included a natural (LEM5159) and an experimental (LEM3323-MIL) MIL-resistant strain (Table 1). Whole genome sequencing explored the genomic basis of MIL resistance of both strains and revealed the presence of a 2 bp-deletion in the *LiMT* gene of LEM3323-MIL leading to an early stop codon and inactivation of the LiMT protein. No mutations in the *LiRos3* gene were found. On the other hand, sequencing of the MIL-resistant LEM5159 clinical isolate revealed mutations in both *LiMT/LiRos3* transporter genes (Table 2). To assess the individual functional role of these mutations, cell transfection experiments were performed whereby transfection of LEM3323-MIL with wild-type *LiMT* resulted in the expression of the LiMT protein and full recovery of MIL-susceptibility (Table 1, Figs 1 and 2). These findings clearly indicate that the mutation in the *LiMT* gene was likely the sole determinant for the acquired resistance. In the natural resistant LEM5159 with both transporter genes showing mutations, re-establishment of full MIL-susceptibility was achieved after transfection with *LiRos3* (Table 1) leading to accumulation of [^14^C]MIL (Fig 1) and expression of both LiRos3 and LiMT proteins (Fig 2). These findings indicate that a major defect in the *LiRos3* gene results in loss of functionality of the inward-directed transporter. The contribution of the E926Q substitution in the *LiMT* gene to the level of resistance was negligible since transfection of LEM5159 with an *LiMT* plasmid failed to restore MIL-susceptibility (Table 1). Surprisingly, no expression of the LiMT protein could be detected in both the parental and *LiMT* transfected LEM5159 strain (Fig 2). Moreover, introduction of an *LiMT*^*E926Q*^*GFP* plasmid containing the LEM5159 characteristic LiMT mutation into a Δ*LdMT* strain was equally efficient as an *LiMTGFP* plasmid to reconstitute MIL-susceptibility, hereby reinforcing that the non-synonymous mutation in LiMT does not add to the overall resistance level of LEM5159 (Table 1, Fig 2). This fully supports the functional characterization of the translocation machinery for which it was shown that the LdMT and LdRos3 are part of the same inward phospholipid transporter [15]. LdMT, a member of the P4 subfamily of P-type ATPases, is involved in phospholipid translocation across the plasma membrane (PM) of *Leishmania* parasites together with its β-subunit LdRos3, a member of the Lem3/CDC50 family [14,38]. LdMT and LdRos3 are required for the translocation activity and normally become localized in the PM, but are retained inside the endoplasmic reticulum in the absence of the other protein or when inactivating point mutations are introduced in LdMT [38]. The presence of a functional LiRos3 in the *LiMT*-transfected LEM3323-MIL and the *LiRos3*-transfected LEM5159 allowed the functional expression of LiMT, while the *LiMT*-transfected LEM5159 showed no expression of LiMT due to lack of a functional *LiRos3* protein (Fig 2). However, the absence of LiMT protein expression could be restored upon the introduction of the *LiRos3* gene in both the LEM5159 strain and the *ΔLiRos3* strain, endorsing the pivotal role for the Ros3 subunit for LiMT protein expression. However, systematic sequencing of clinical *L*. *donovani* isolates from patients showing a relapse after MIL treatment did not (yet) reveal any mutations in LdMT and LdRos3 (*Imamura*, *unpublished results*). Extrapolation of genetic markers identified in experimentally-induced parasites to the field may not be straightforward due to changing environments and host immunity [39]. In addition to the findings of Cojean et al, 2012, our results demonstrate that different genes can be involved in the dysfunctionality of LiRos/LiMT of which both can be affected by deletions, point mutations or frame shifts. Hence, defining suitable markers for MIL-resistance may still prove to be very difficult and would imply that a genetic diagnostic test would have to rely on full length sequencing of both LiMT and LiRos, which appears practically unfeasible at large-scale. Efflux rates were not measured as there were no indications that efflux is involved in the strains characterized in this study, particularly given the fact that reconstitution of resistant strains with the inward transporter is sufficient to fully restore susceptibility. Literature data suggest that drug efflux may only be relevant in conditions of high MIL-uptake, for example in cancer cells [40].

Interestingly, our data showed that the experimental amastigote model correlated fairly well with the *in vivo* situation, hereby supporting the use of LiMT and LiRos3 transporter genes as relevant molecular markers of MIL-resistance in the field. Whole genome sequencing of an extended selection of clinical isolates derived from MIL-treatment failures should give more valuable information about the appearance of particular gene mutations in relation to the occurrence of treatment relapses. On the other hand, treatment failure has a more multifactorial origin and should not solely be linked to drug resistance [41,42]. For example, *L*. *donovani* isolates from cured and relapsed patients in the Indian subcontinent showed similar MIL-susceptibility [6] while those of post-kala-azar dermal leishmaniasis (PKDL) patients showed reduced *in vitro* susceptibility [10,43] suggesting the involvement of different mechanisms in treatment failure. Rai *et al*. reported an association between the increased infectivity of *L*. *donovani* parasites with MIL-relapse of VL patients, indicating the importance to assess other phenotypes than drug susceptibility when characterizing parasites from relapse patients [44]. Moreover, it is recommended to combine the genomic screening of clinical isolates with functional studies to validate the exact contribution of identified mutations in the acquisition of resistance. Metabolomic studies may further shed light on the very complex nature of MIL-resistance, treatment failure and relapse [45–46].

In conclusion, this study is the first to explore the genomic and functional molecular basis of MIL-resistance in two *L*. *infantum* strains that definitively developed resistance in the intracellular amastigote stage. The natural resistant patient isolate LEM5159 and the experimentally *in vitro* selected LEM3323-MIL both showed changes in the MIL-translocation machinery leading to the acquisition of the full blown MIL-resistance phenotype, providing compelling evidence that the *in vitro* amastigote resistance selection model could be a good proxy of what may happen in the *in vivo* field situation. A defect in the inward translocation machinery through inactivation of the LiMT/LiRos3 proteins remains the main mechanism of MIL-resistance [47] and supports current literature findings obtained for promastigotes. It is evident that similar work needs to be carried out with *L*. *donovani* strains, but ongoing *in vitro* and *in vivo* laboratory work already indicates that MIL-resistance selection in *L*. *donovani* appears much more slowly requiring a greater number of passages [12,17] and that the availability of MIL-resistant clinical isolates certainly remains a critical issue.

## Supporting Information

S1 Fig **Somy of the 36 chromosomes of *L*. *infantum*, inferred by whole genome sequencing**: **(A)** Comparison between the parent LEM3323 and the experimentally derived MIL-resistant LEM3323-MIL; **(B)** Comparison between LEM3049 and the natural MIL-resistant isolate LEM5159. The error bars indicate the ploidy standard deviation within individual chromosomes.
(TIF)

S2 Fig Confocal images of the intracellular distribution of BODIPY-MIL in *L*. *infantum* promastigotes. Parasites were incubated with 2 µM BODIPY-MIL for 1h. (a) MIL-susceptible LEM3323; (b) MIL-resistant strains LEM3323-MIL and (c) LEM5159. Excitation/emission wavelengths were 529/536 nm for BODIPY-labelled MIL.
(TIF)

S3 Fig DAPI staining and uptake of BODIPY-MIL by intracellular amastigotes. (a) MIL-susceptible LEM3323, (b) *in vitro* MIL-resistant LEM3323-MIL and (c) MIL-resistant clinical isolate LEM5159. The intracellular amastigotes appear as small blue spots while the PMM nucleus is a big blue spot. The wild-type strain shows a clear association between the DAPI spot and the green fluorescence. Excitation/emission wavelengths were 529/536 nm for BODIPY-labelled MIL and 365/445 nm for DAPI.
(TIF)

S1 Table Whole-genome sequencing of *L*. *infantum* isolates. List of SNPs or indels in coding regions of LEM3323 and LEM3323-MIL that differed between these two isolates; 0/0, homozygous reference; 0/1, heterozygous altered, 1/1, homozygous altered. The variants were called against the *L*. *infantum* JPCM5 reference genome.
(DOCX)
