# Supplementary figures and images for: Genomic and Molecular Characterization of Miltefosine Resistance in Leishmania infantum Strains with Either Natural or Acquired Resistance through Experimental Selection of Intracellular Amastigotes
Source: PLoS One. 2016 Apr 28;11(4):e0154101. doi: 10.1371/journal.pone.0154101 (PMC4849676; doi:10.1371/journal.pone.0154101)

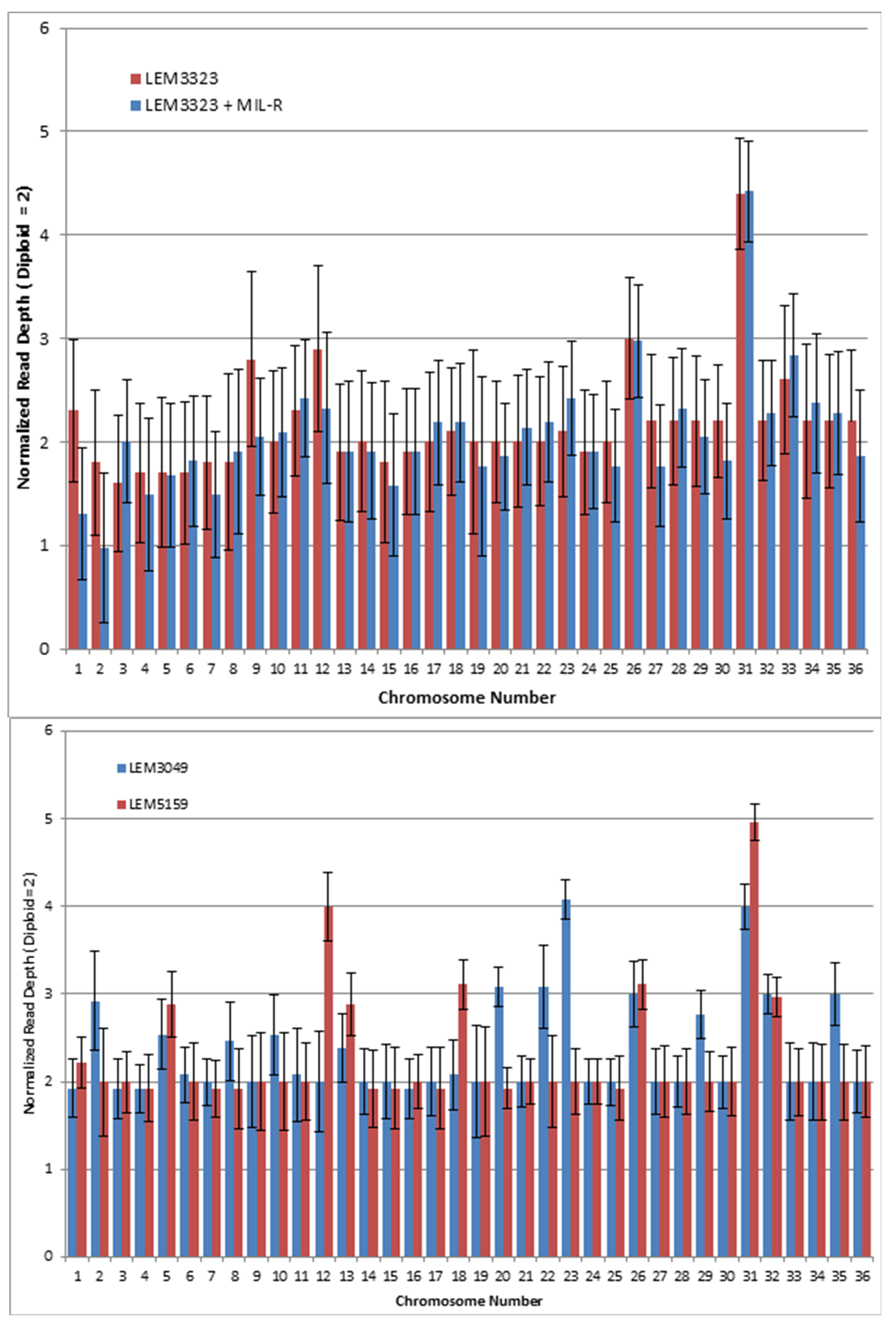

Supplement: S1 Fig — Somy of the 36 chromosomes of L. infantum, inferred by whole genome sequencing: (A) Comparison between the parent LEM3323 and the experimentally derived MIL-resistant LEM3323-MIL; (B) Comparison between LEM3049 and the natural MIL-resistant isolate LEM5159. The error bars indicate the ploidy standard deviation within individual chromosomes. (TIF) [file pone.0154101.s001.tif]

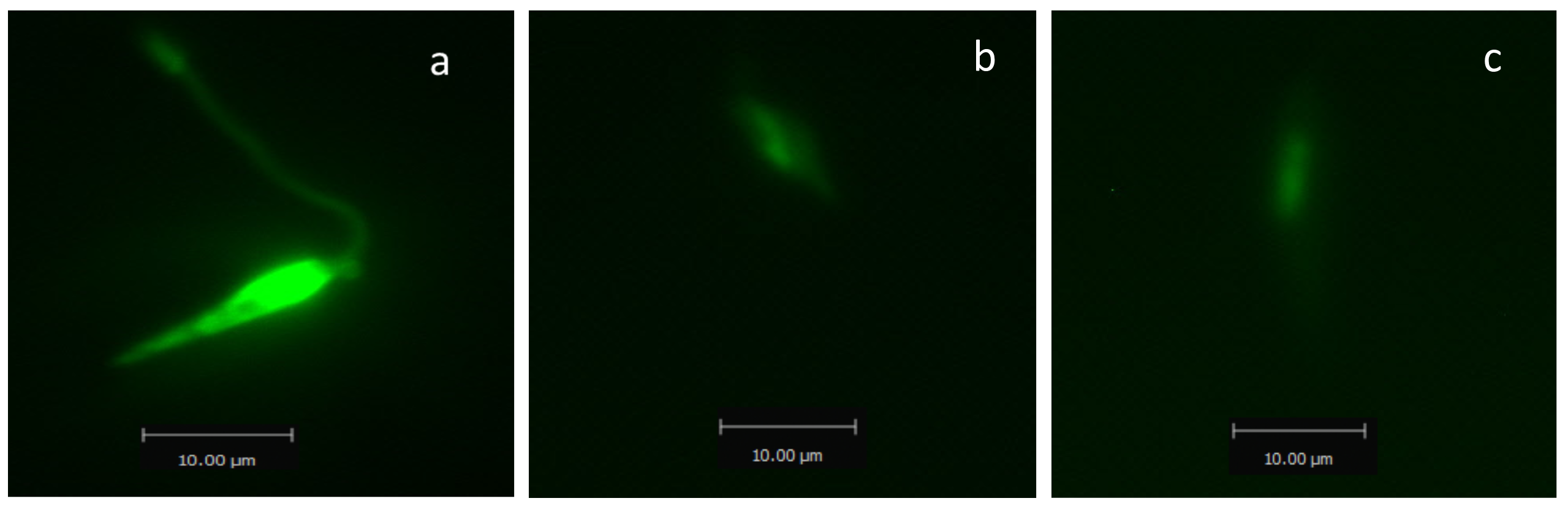

Supplement: S2 Fig — Parasites were incubated with 2 µM BODIPY-MIL for 1h. (a) MIL-susceptible LEM3323; (b) MIL-resistant strains LEM3323-MIL and (c) LEM5159. Excitation/emission wavelengths were 529/536 nm for BODIPY-labelled MIL. (TIF) [file pone.0154101.s002.tif]

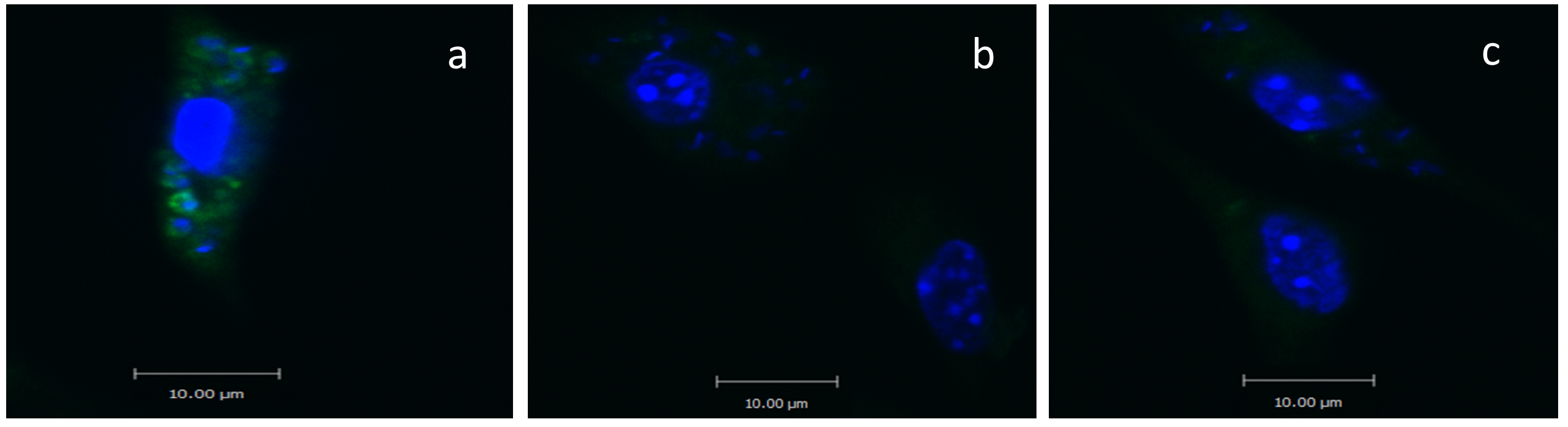

Supplement: S3 Fig — (a) MIL-susceptible LEM3323, (b) in vitro MIL-resistant LEM3323-MIL and (c) MIL-resistant clinical isolate LEM5159. The intracellular amastigotes appear as small blue spots while the PMM nucleus is a big blue spot. The wild-type strain shows a clear association between the DAPI spot and the green fluorescence. Excitation/emission wavelengths were 529/536 nm for BODIPY-labelled MIL and 365/445 nm for DAPI. (TIF) [file pone.0154101.s003.tif]
