# Supplementary material for: Genomic and Molecular Characterization of Miltefosine Resistance in Leishmania infantum Strains with Either Natural or Acquired Resistance through Experimental Selection of Intracellular Amastigotes
Source: PLoS One. 2016 Apr 28;11(4):e0154101. doi: 10.1371/journal.pone.0154101 (PMC4849676; doi:10.1371/journal.pone.0154101)
Supplement: S1 Table — List of SNPs or indels in coding regions of LEM3323 and LEM3323-MIL that differed between these two isolates; 0/0, homozygous reference; 0/1, heterozygous altered, 1/1, homozygous altered. The variants were called against the L. infantum JPCM5 reference genome. (DOCX) [file pone.0154101.s004.docx]

| **Gene name** | **Position** | **Ref** | **Alt** | **LEM3323** | **LEM3323-MIL** | **Annotation** |
| --- | --- | --- | --- | --- | --- | --- |
| LinJ.13.1590 | 619572 | CCACA | CCA | 0/0 | 1/1 | Phospholipid transporting ATPase1 |
| LinJ.36.5160 | 1902086 | G | A | 0/0 | 0/1 | Translation initiation factor 2 subunit |
| LinJ.32.2460 | 912664 | C | T | 1/1 | 0/1 | Hypoth. protein |
| LinJ.28.0650 | 231253 | C | T | 0/0 | 0/1 | Dynein heavy chain |
| LinJ.29.0740 | 263282 | G | C | 0/1 | 0/0 | Hypoth. protein |
| LinJ.30.1820 | 647491 | A | C | 0/0 | 0/1 | Splicing factor 3a |
| LinJ.30.3480 | 1261850 | A | G | 0/0 | 0/1 | Protein mkt1 |
